# Educator Toolkits on Second Victim Syndrome, Mindfulness and Meditation, and Positive Psychology: The 2017 Resident Wellness Consensus Summit

**DOI:** 10.5811/cpcem.2017.11.36179

**Published:** 2018-02-12

**Authors:** Arlene S. Chung, Jon Smart, Michael Zdradzinski, Sarah Roth, Alecia Gende, Kylie Conroy, Nicole Battaglioli

**Affiliations:** * Icahn School of Medicine at Mount Sinai, Department of Emergency Medicine, New York, New York; † University of Texas Health Science Center San Antonio, Department of Emergency Medicine, San Antonio, Texas; ‡ Emory University School of Medicine, Department of Emergency Medicine, Atlanta, Georgia; § Kingman Regional Medical Center, Department of Emergency Medicine, Kingman, Arizona; ¶ University of Iowa Hospitals and Clinics, Department of Emergency Medicine, Iowa City, Iowa; || University of Arizona, Department of Emergency Medicine, Tucson, Arizona; # The Mayo Clinic, Department of Emergency Medicine, Rochester, Minnesota

## Abstract

**Introduction:**

Burnout, depression, and suicidality among residents of all specialties have become a critical focus of attention for the medical education community.

**Methods:**

As part of the 2017 Resident Wellness Consensus Summit in Las Vegas, Nevada, resident participants from 31 programs collaborated in the Educator Toolkit workgroup. Over a seven-month period leading up to the summit, this workgroup convened virtually in the Wellness Think Tank, an online resident community, to perform a literature review and draft curricular plans on three core wellness topics. These topics were second victim syndrome, mindfulness and meditation, and positive psychology. At the live summit event, the workgroup expanded to include residents outside the Wellness Think Tank to obtain a broader consensus of the evidence-based toolkits for these three topics.

**Results:**

Three educator toolkits were developed. The second victim syndrome toolkit has four modules, each with a pre-reading material and a leader (educator) guide. In the mindfulness and meditation toolkit, there are three modules with a leader guide in addition to a longitudinal, guided meditation plan. The positive psychology toolkit has two modules, each with a leader guide and a PowerPoint slide set. These toolkits provide educators the necessary resources, reading materials, and lesson plans to implement didactic sessions in their residency curriculum.

**Conclusion:**

Residents from across the world collaborated and convened to reach a consensus on high-yield—and potentially high-impact—lesson plans that programs can use to promote and improve resident wellness. These lesson plans may stand alone or be incorporated into a larger wellness curriculum.

## INTRODUCTION

Burnout, depression, and suicidality among residents across all specialties have become a critical focus of attention for the medical education community. Prevalence studies have revealed rates of burnout among residents to be as high as 76%, as measured by the Maslach Burnout Inventory (MBI).^1^ Residents suffering from burnout have a higher risk than their peers of developing depression, anxiety, and substance-abuse problems.^2^ Even more alarmingly, up to 9.4% of fourth-year medical students and interns reported having suicidal thoughts.^3^ These numbers are borne out in the estimated 400 physicians who commit suicide each year.^4^

In response to these findings, the Accreditation Council for Graduate Medical Education (ACGME) approved major changes to the Common Program Requirements to begin in July 2017. In section VI.C, residency programs are mandated to educate residents and faculty members in the identification of burnout, depression, and substance abuse and to implement curricula that encourage their optimal wellbeing.^5^ However, the ACGME has yet to provide residency programs with concrete guidelines for the creation of wellness curricula to adequately address this mandate.

Many residency programs have already implemented some form of wellness training for their residents. Unfortunately, evidence supporting the efficacy of these interventions is sparse and often limited to single institutions and small sample sizes.^6^,^7^ Nor has the medical education community reached an agreement on the best method of identifying relevant and high-impact wellness topics for residents, or understanding the optimal method for delivery and dissemination of information.

## METHODS

From October 3, 2016, to May 14, 2017, members of the Wellness Think Tank communicated through a shared online platform (#Slack) to discuss the strengths and weaknesses of the wellness programs at their respective training sites. The Think Tank is a virtual community of practice, hosted by a medical education organization Academic Life in Emergency Medicine, which is comprised of 142 emergency medicine (EM) residents from 100 different training programs in North America. Multiple residents noted a haphazard and ineffective approach to teaching wellness topics, which they attributed primarily to a lack of shared knowledge between residency programs. Residents voiced a clear need for more widely-shared lesson plans that focus on the development of practical skills fostering personal wellness.

In preparation for the 2017 Resident Wellness Consensus Summit (RWCS) in-person event on May 15, 2017, an Educator Toolkit working group was created. Using a group consensus process, the residents of the Wellness Think Tank selected and agreed upon three high-yield topics that would benefit educators as an evidence-based, robust resource: second victim syndrome (SVS), mindfulness and meditation, and positive psychology. Each toolkit was designed using Kern’s six-step model of curriculum design, active teaching techniques, and accountability to increase engagement. Representing 20 different programs, 8, 16, and 17 residents participated in the development of the SVS, mindfulness and meditation, and positive psychology educator toolkits, respectively. Two faculty members (A.C., N.B.) trained in educational theory, one with a master’s degree in medical education, provided oversight.

**Population Health Research Capsule**

What do we already know about this issue?

Programs are now required to provide wellness education for residents. However, no concrete implementation guidelines currently exist for educators.

What was the research question?

What would happen if residents from across the world collaborated and reached consensus on strategies to help improve resident wellness?

What was the major finding of the study?

The outcome of the collaboration was the development of three comprehensive educator toolkits containing resources, reading materials, and lesson plans.

How does this improve population health?

These toolkits address three topics – second victim syndrome, meditation, and positive psychology – identified by residents as important to their education, and provide concrete guidelines for educators to implement curricula.

Twenty-two resident members of the Wellness Think Tank and 22 additional residents attended the live RWCS event. These residents represented 31 EM residency programs located in three different countries. Five faculty members facilitated the event. Members of the Educator Toolkit working group presented their drafts to the RWCS consensus group for review. There, participants discussed the proposed topics, learning objectives, and teaching techniques for each of the three topics. Following the RWCS, each educator toolkit was further refined based on the feedback, which resulted in the final versions presented here.

## RESULTS

Three educator toolkit resources were developed through a consensus achieved among residents in the Wellness Think Tank and the live RWCS event over an eight-month period. The three topics include SVS, mindfulness and meditation, and positive psychology. These resources are open access and available in Appendices A–C.

### Second Victim Syndrome

A phenomenon growing in national awareness,^8^–^10^ SVS is commonly defined as feelings of guilt, inadequacy, or incompetence following an unexpected, negative patient outcome. Commonly manifesting as anxiety, depression or shame, it often goes unrecognized. It is likely that most healthcare providers experience symptoms of SVS at least once in their careers and the emotional “wear-and-tear” may contribute to burnout, the decision to depart from clinical medicine, or even suicide.^8^,^11^,^12^ Victims of SVS may require assistance from mentors, colleagues, or mental health professionals to cope with the frequently intense, negative personal and professional ramifications of the experience.^13^,^14^ Awareness of the existence of SVS is critical for residents and faculty so that they may develop strategies to mitigate the negative effects in both themselves and their colleagues.

Despite its relevance across specialties, and especially EM, no published residency curricula discuss SVS. Our toolkit aims to fill that education gap to ensure that residents are prepared for the emotional and professional toll from inevitable negative patient outcomes that will occur during their careers.

To maximize learner engagement and provide flexibility for residency programs, this toolkit includes four “mini-modules” using a flipped-classroom approach (Appendix A). Each module consists of a pre-reading assignment followed by a 20-minute group discussion. Pre-reading establishes a basic knowledge base for the learners and encourages personal reflection prior to the classroom session. Group discussions aid in the practical incorporation of that knowledge into the residents’ practice. It is recommended that the four modules be spaced over several months to maximize retention of the material via spaced repetition.^16^ The mini-modules may also be combined into a single 60–90 minute session to accommodate didactic conference schedules.

The first module exposes learners to the concept of SVS, as well as potential stages of recovery. This module emphasizes establishing a foundation of knowledge pertaining to SVS, laying the groundwork for later modules to introduce practical tools and concepts for coping with and preventing SVS. The second module describes a method to help recognize SVS in colleagues. Residents and faculty are encouraged to help colleagues identify when they are suffering from SVS and to help create an appropriate follow-up plan. In addition, a method for performing a “hot debriefing” is described, which occurs immediately following a significant mistake or negative patient outcome. The third module serves to make learners aware of resources that are available at their individual institution and encourages learners to access them prior to being affected by SVS. Finally, the fourth module focuses on department-wide prevention of SVS through culture change and the use of routine, group debriefings following difficult resuscitations.^17^–^21^ A standardized post-resuscitation debriefing template is introduced.

### Mindfulness and Meditation

Mindfulness is the practice of purposeful and nonjudgmental attentiveness to one’s own experience, thoughts, and feelings. Meditation is a technique for resting the mind and attaining a state of consciousness that is distinctly different from the normal waking state. A regular practice of meditation can provide a lasting sense of mindfulness that lasts throughout the day. Both mindfulness and meditation have become more mainstream and socially acceptable ways to manage stress and increase productivity. Within the field of medicine, research has shown that being mindful or developing a meditation practice improves job satisfaction and decreases burnout.^22^ Multiple studies have demonstrated benefits to mindfulness and meditation, such as increased empathy, life satisfaction and self-compassion, and decreased anxiety, rumination, and burnout, and decreased cortisol levels.^23^ Because EM residents stand to benefit tremendously from these effects, we determined that mindfulness and meditation were important as a wellness toolkit for educators.

Although mindfulness and meditation have become more integrated into some medical schools, these concepts are not frequently found in residency training programs.^24^ We developed a mindfulness and meditation lesson plan to address this gap. Our educator toolkit consists of three 30-minute modules and a longitudinal, guided group meditation practice designed to span several months or an academic year.

The two initial modules outline and define meditation, as well as describe how to start a meditation practice. These modules include an opportunity to practice meditating as a group, an invitation to start an individual practice, and a chance to discuss barriers to practice. Ideally, these modules would be offered during the first month of the academic year, separated by one to four weeks. Following the second module, a longitudinal, guided meditation practice should be incorporated at regular intervals (weekly, monthly or quarterly) throughout the residency conference schedule. The final module should be implemented toward the end of the academic year following the longitudinal meditations. This module provides the residents a forum to debrief and reflect on their practices of meditation and mindful thinking, cultivated throughout the year. It also serves as a chance to consider and evaluate how meditation and mindfulness have impacted individual residents, the residency program, and the department.

### Positive Psychology

Positive psychology is the conscious participation in acts to improve wellbeing by creating and nurturing positive feelings, thoughts, and behaviors. In contrast to traditional psychology, which focuses on mitigating illness, positive psychology focuses on the strengths that allow individuals to thrive.^25^ Use of positive psychology interventions has been shown to improve wellbeing and decrease depressive symptoms.^26^–^28^ Positive psychology interventions can serve as a useful tool to improve team dynamics and success in stressful situations such as trauma resuscitations.^29^ Practicing gratitude, positive self-talk, and intentional acts of kindness have the potential to make emotionally difficult shifts more tolerable and improve physician-patient interactions.

Despite the literature describing the benefits of positive psychology, similar to SVS and mindfulness and meditation, we found no described use of a positive psychology curriculum for residents. To address this gap, we developed a flexible and easy-to-implement positive psychology toolkit, focusing on two positive psychology principles, PERMA (Positive Emotions, Engagement, Relationships, Meaning, Accomplishment) and BTSF (Breathe, Talk, See, Focus). Although these positive psychology principles can be taught together as a two-part, positive psychology lesson plan, each can also be given as a stand-alone session.

PERMA is an evidence-based model for wellbeing that can help residents to more fully engage with their work and thrive in their careers.^30^ The PERMA toolkit includes a slide set presentation, brainstorming period, and both paired and group discussion over a period of 50 minutes. The slide set provides a basic introduction to positive psychology, followed by a more detailed description of the PERMA model. Interactive audience participation during the slide presentation is encouraged. A brainstorming activity in pairs and in a larger group follows the slide presentation. The session concludes with a “commitment to act,” an exercise in which the learners write down one specific thing that they plan to do differently based on their participation.

BTSF is a skill that can be quickly taught to residents and used in a wide variety of settings. This technique helps an individual cope with an acute stressor by employing one or all four of the following: (1) tactical or box breathing; (2) positive self-talk; (3) visualizing success; and (4) stating or intentionally thinking a specific focus word to hone attention.^31^ Similar to the PERMA lesson plan, the BTSF session includes a slide set presentation, active group discussion, and an acute stressor exercise for participants to practice BTSF in a simulated stressful environment over a period of 45 minutes. The slide set specifically describes each of the components of BTSF and concludes with a tactical breathing exercise. The conclusion of the BTSF lesson plan is an acute stressor exercise. We suggest using the children’s game, Operation (© Hasbro), or other similar game to simulate stress while practicing the BTSF model. The lesson plan also concludes with a “commitment to act” exercise.

## DISCUSSION

The 2017 Resident Wellness Consensus Summit was convened with the ultimate mission to empower EM residents from around the world to lead efforts aimed at decreasing burnout, depression, and suicidality during residency and to increase resident wellbeing. Leading up to the event, many residents collaborated in the Wellness Think Tank over an eight-month period to conduct much of the consensus event prework. Specifically our Educator Toolkit working group focused on developing three widely-applicable, high-yield lesson plans for EM residency programs on the topics of SVS, mindfulness and meditation, and positive psychology.

The lesson plans may stand alone or be incorporated into a larger wellness program. The three toolkits differ in length, scope, and duration for the individual sessions. This design provides greater flexibility for residency programs to schedule into their existing training curriculum. For example, programs with limited time or resources may find a single 45-minute session on positive psychology easier to incorporate than a year-long curriculum that includes classroom sessions and monthly guided meditations, as described in the mindfulness and meditation toolkit.

In an effort to address widespread burnout and unwellness, our goal is for these three topics to be widely covered and implemented by residency programs through the use of these templated lesson plans. Each toolkit provides instruction on practical skills training that can be used on a daily basis, both within and outside of the emergency department. Next steps include measuring the effects of these lesson plans on resident satisfaction, learning, behavior change, and ultimately patient outcomes, as well as burnout, resilience, and job satisfaction.

## CONCLUSION

The 2017 Resident Wellness Consensus Summit was a unique and novel consensus event, particularly because its main audience was resident stakeholders. As a product of this wellness summit, three comprehensive lesson plans were developed for resident education on the topics of second victim syndrome, mindfulness and meditation, and positive psychology. These educator toolkit resources were developed through the consensus and collaboration of residents in the Wellness Think Tank and those who attended the live RWCS event.
